# New Immunological Insights into Bisphosphonate-Related Osteonecrosis of the Jaw: A Multidimensional Reappraisal from Molecular Switches to Clinical Phenotypes

**DOI:** 10.3390/cimb48080835

**Published:** 2026-08-17

**Authors:** Yao Li, Shengao Qin, Jiaqi Wang, Zhaochen Shan, Jiaxin Song, Haitong Zhang, Wen Pan, Zhao Zhu

**Affiliations:** 1 Department of Oral and Maxillofacial Surgery, School of Stomatology, Capital Medical University, Beijing 100070, China; yaoli034@163.com (Y.L.); shengaoqin123@163.com (S.Q.); shanzhch629@163.com (Z.S.); songjx815@163.com (J.S.); m13126716716@163.com (H.Z.); 2 Department of Oral Medicine, School of Stomatology, Capital Medical University, Beijing 100070, China; jiaqiwang5923@163.com

**Keywords:** bisphosphonates, bisphosphonate-related osteonecrosis of the jaw, osteoimmune microenvironment, immune remodeling, targeted therapy

## Abstract

Bisphosphonates (BPs) are cornerstone therapeutic agents for the management of metabolic bone disorders and skeletal complications associated with malignant tumors. However, their long-term administration is accompanied by the risk of developing bisphosphonate-related osteonecrosis of the jaw (BRONJ). In recent years, the pathogenesis of BRONJ has remained a major focus at the intersection of osteoimmunology and oral medicine. This review systematically summarizes the latest advances in the understanding of BRONJ pathogenesis, including the direct disruption of osteoclast differentiation and bone remodeling coupling by bisphosphonates, the systemic reprogramming of immune cell subsets and inflammatory signaling pathways, the multifaceted suppression of angiogenesis and the endothelial–stromal–immune axis, and the toxic impairment of oral soft tissue repair capacity. Building upon these mechanistic insights, we further discuss the modifying and synergistic effects of local oral risk factors, microbial dysbiosis, systemic comorbidities, and genetic susceptibility on the initiation and progression of BRONJ. Furthermore, emerging therapeutic strategies targeting these pathogenic mechanisms are highlighted, including restoration of small GTPase prenylation through modulation of metabolic pathways, reconstruction of T-cell subset homeostasis via immunomodulatory approaches, enhancement of local vascularization through pro-angiogenic interventions, and preservation of soft tissue barrier integrity. Collectively, this review provides a comprehensive and multidimensional overview of the pathological landscape of BRONJ and offers a theoretical framework for clinical risk stratification and the development of personalized prevention and treatment strategies.

## 1. Introduction

Bisphosphonates (BPs) are synthetic analogs of inorganic pyrophosphate in which the oxygen atom within the P-O-P backbone is replaced by a carbon atom, forming a characteristic P-C-P structure. The R1 side chain attached to the central carbon atom enhances calcium-binding affinity, whereas the R2 side chain primarily determines antiresorptive potency and pharmacological activity [1]. Based on the presence or absence of nitrogen within the R2 side chain, bisphosphonates are classified into nitrogen-containing bisphosphonates (N-BPs), such as alendronate, pamidronate, and zoledronic acid, and non-nitrogen-containing bisphosphonates (non-N-BPs), including etidronate and tiludronate [2]. Owing to their potent inhibitory effects on osteoclast-mediated bone resorption, bisphosphonates have been widely applied in the treatment of osteoporosis [3], the reduction in skeletal complications associated with metastatic prostate cancer [4], adjuvant therapy and recurrence prevention in breast cancer [5], short-term pain management in complex regional pain syndrome [6], and the suppression of bone metastases in advanced breast cancer [7].

Medication-related osteonecrosis of the jaw (MRONJ) is a severe jawbone disorder associated with antiresorptive or immune-modulating agents. It is clinically characterized by exposed necrotic bone, or bone that can be probed through an intraoral or extraoral fistula, persisting for more than eight weeks in the absence of a history of radiation therapy to the jaws or metastatic disease involving the jawbones [8]. Cases specifically induced by bisphosphonates are referred to as bisphosphonate-related osteonecrosis of the jaw (BRONJ). Clinical evidence indicates that patients receiving bisphosphonate therapy exhibit a 3.45-fold higher risk of developing osteonecrosis of the jaw compared with non-users [9]. Moreover, the risk is substantially greater with intravenous administration than with oral therapy and increases progressively with treatment duration [10].

The pathogenesis of BRONJ may extend far beyond a simple suppression of bone turnover. Increasing evidence suggests that BRONJ represents a multifactorial cascade initiated by immune dysregulation and culminating in the collapse of local tissue homeostasis and microenvironmental integrity. Against the background of impaired osteoclast function and defective angiogenesis, immune suppression and aberrant inflammatory signaling have emerged as central drivers of disease progression. In this context, the present review systematically examines the differential effects of bisphosphonates on both innate and adaptive immune compartments, including γδ T-cell depletion, disruption of the regulatory T cell (Treg)/T helper 17 (Th17) balance, alterations in natural killer (NK) cell cytotoxicity, skewing of macrophage M1/M2 polarization, neutrophil dysfunction, impaired dendritic cell antigen presentation, and the expansion of myeloid-derived suppressor cells (MDSCs). At the molecular level, particular attention is devoted to the inflammatory amplification mechanisms mediated by the IL-36α signaling pathway and the TNF-α/TNFR1 axis.

**Figure 1:**
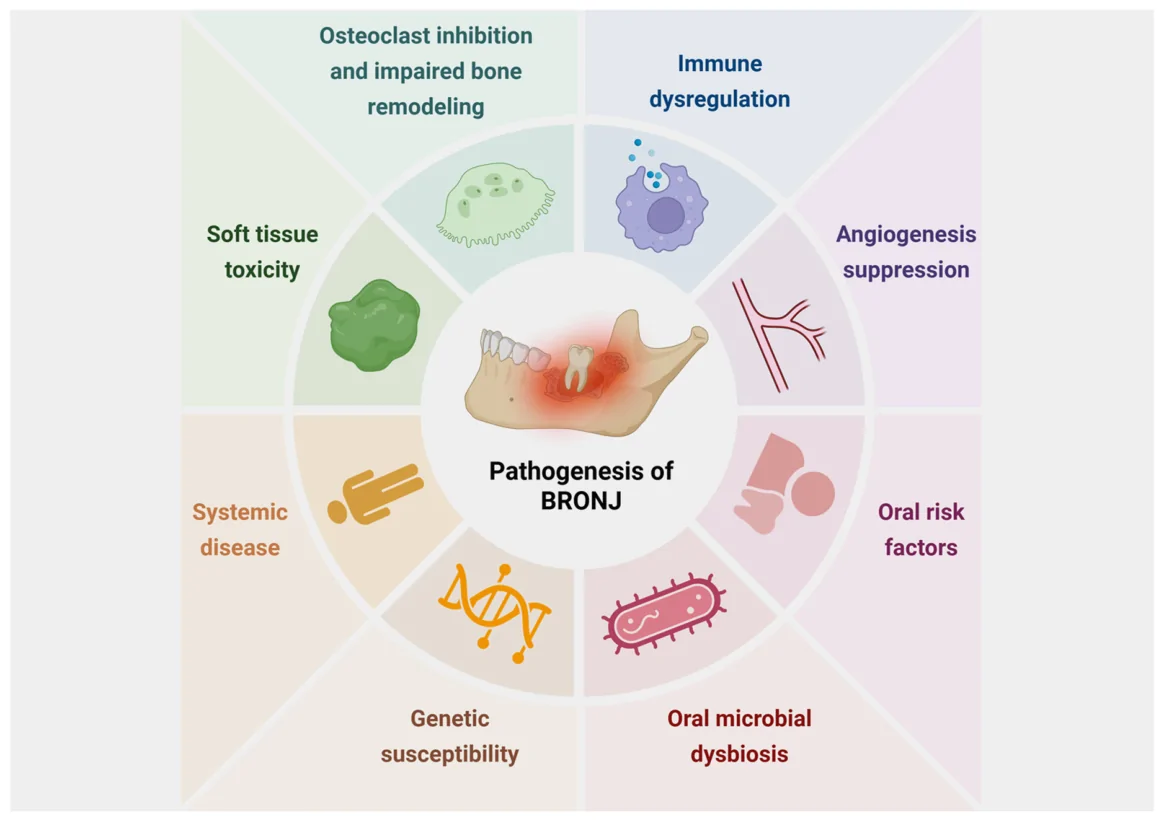
The Multifactorial Pathogenic Landscape and Integrated Etiologies of BRONJ. Created in BioRender. Pan, W. (2026) https://BioRender.com/wk2vtf2.

**Figure 2:**
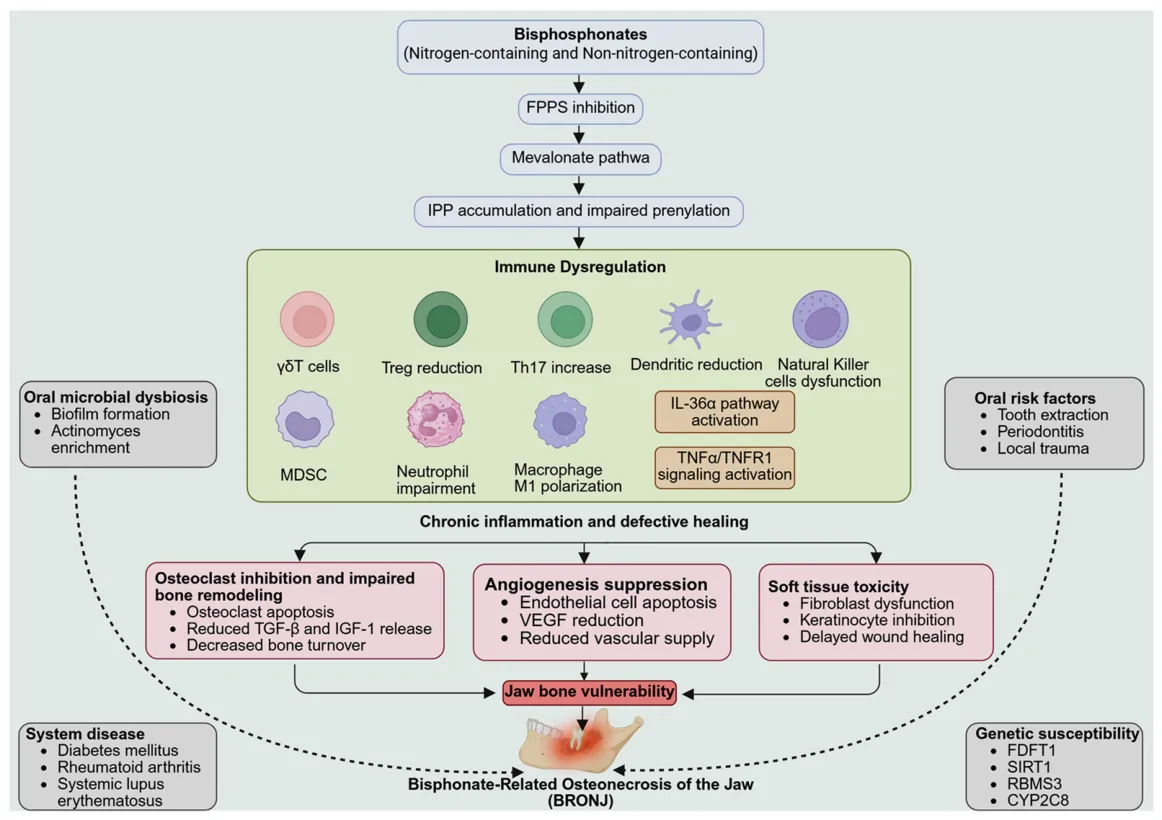
The Central Pathogenic Network and Cellular Cross-talk in BRONJ. This schematic illustration integrates the major pathogenic mechanisms of nitrogen-containing bisphosphonates (N-BPs) within the intraoral microenvironment, including impaired bone remodeling and angiogenesis, immune dysregulation and persistent inflammation, and soft-tissue injury and microbial dysbiosis. N-BPs inhibit farnesyl diphosphate synthase (FDPS) within the mevalonate pathway, promoting osteoclast dysfunction and apoptosis and establishing a low-turnover state, while concurrently suppressing angiogenesis and contributing to local vascular insufficiency. Bisphosphonates (BPs) also disrupt immune cell functions, including those of γδ T cells, regulatory T (Treg) cells, neutrophils, and dendritic cells, while promoting T helper 17 (Th17) cell expansion and macrophage polarization toward the pro-inflammatory M1 phenotype, thereby sustaining an inflammatory microenvironment. In addition, BPs exert direct cytotoxic effects on oral keratinocytes and fibroblasts, impairing mucosal wound healing and facilitating persistent microbial inflammatory stimulation. Created in BioRender. Pan, W. (2026) https://BioRender.com/kiirql0.

Building upon this mechanistic framework, the scope of this review extends beyond the question of “how BRONJ develops” to address “how BRONJ may be therapeutically targeted”. We summarize emerging intervention strategies directed at key nodes within these immune regulatory networks and discuss their translational potential, current limitations, and challenges in clinical implementation. By integrating advances in osteoimmunology, molecular pathology, and therapeutic development, this review aims to provide a comprehensive perspective on the immunopathogenesis of BRONJ and to identify potential avenues for precision prevention and individualized treatment. The multifaceted and intertwined etiologies contributing to the development of BRONJ are conceptually consolidated into a holistic multi-sector wheel diagram (Figure 1). The complex cellular cross-talk and molecular signaling networks driving bisphosphonate-induced jaw osteonecrosis are synergistically integrated and visualized in Figure 2.

## 2. Molecular Basis of Osteoclast Inhibition and Its Osteoimmunological Implications

BPs function as a “bone hook”, enabling high-affinity binding to hydroxyapatite (HAP) through the formation of a tridentate ligand complex [11]. During bone resorption, BPs are released from HAP and internalized by osteoclasts in the form of calcium complexes, subsequently entering the cytoplasm and acting on their intracellular targets [12]. Nitrogen-containing and non-nitrogen-containing BPs exert distinct effects on osteoclasts. Non-nitrogen-containing BPs primarily interact with class II aminoacyl-tRNA synthetases, and their toxic metabolite AppCCl_2_p induces functional ATP depletion and inhibits mitochondrial ADP/ATP translocase activity [13,14]. Furthermore, the accumulation of non-hydrolyzable metabolites suppresses ANT activity, triggering alterations in mitochondrial permeability and caspase-3-mediated apoptosis [15].

The mevalonate pathway begins with the conversion of acetyl-CoA into mevalonate catalyzed by 3-hydroxy-3-methylglutaryl-CoA reductase (HMGCR), followed by phosphorylation and decarboxylation reactions that generate isopentenyl pyrophosphate (IPP) and dimethylallyl pyrophosphate (DMAPP). Farnesyl diphosphate synthase (FDPS) catalyzes the condensation of IPP and DMAPP to produce farnesyl pyrophosphate (FPP), which is subsequently converted into geranylgeranyl pyrophosphate (GGPP) by geranylgeranyl pyrophosphate synthase (GGPPS). FPP and GGPP are not only precursors for the biosynthesis of cholesterol, coenzyme Q10 (CoQ10), and heme A, but also essential donors for the prenylation of small GTPases [16,17,18].

Nitrogen-containing bisphosphonates target FDPS and inhibit the mevalonate pathway, thereby reducing the production of FPP, GGPP, and downstream metabolites [19,20]. Their effects on osteoclasts can be summarized in three aspects. First, depletion of FPP and GGPP prevents the prenylation of small GTPases, including members of the Ras, Rho, and Rab families, thereby impairing their localization to the cell membrane [11,21]. Inactivation of the Rho family disrupts the formation of the actin ring and sealing zone, whereas Rab family inactivation interferes with vesicular trafficking and ruffled border formation. Inactivation of Ras family proteins abolishes survival signaling and induces apoptosis [22,23]. Notably, prenylation of small GTPases is not unique to osteoclasts. T-cell receptor signaling, NK cell cytotoxic activity, and macrophage phagocytosis all depend on this modification. Therefore, depletion of FPP and GGPP exerts systemic disruptive effects on the osteoimmune network that extend beyond osteoclast dysfunction. Second, blockade of the mevalonate pathway leads to marked intracellular accumulation of IPP. IPP combines with AMP to form the ATP analog ApppI, which enters mitochondria and inhibits ANT, thereby disrupting mitochondrial function and inducing apoptosis [24,25,26]. Importantly, IPP is not only a toxic metabolite in osteoclasts but also a phosphoantigen recognized by the γδ T-cell receptor (TCR). This provides the molecular basis by which BPs convert an “osteoclast metabolic crisis” into a “systemic immune signal”, a concept that will be discussed in detail in subsequent sections. Furthermore, Giladi HZ et al. reported that patients receiving high-dose simvastatin showed an increased susceptibility to MRONJ in a clinical study, providing additional evidence that dysregulation of the mevalonate pathway may represent a key molecular event contributing to MRONJ pathogenesis [27]. Third, inhibition of the mevalonate pathway reduces the synthesis of CoQ10 and heme A. CoQ10 deficiency disrupts mitochondrial redox homeostasis, resulting in decreased ATP production, increased oxidative stress, and osteoclast apoptosis [28,29]. In addition, reduced CoQ10 levels suppress receptor activator of nuclear factor κB (RANKL)-induced osteoclastogenesis [30]. Decreased heme A synthesis, in contrast, impairs the formation of cytochrome c oxidase (complex IV), thereby compromising the integrity of the mitochondrial respiratory chain [29].

Bone remodeling is a dynamic coupling process between bone resorption and bone formation. During bone resorption, osteoclasts release factors such as transforming growth factor-β (TGF-β) and insulin-like growth factor-1 (IGF-1) from the bone matrix, thereby activating osteoblasts and initiating new bone formation [31]. By inducing osteoclast apoptosis, BPs interrupt these coupling signals and consequently suppress bone remodeling. In addition, BPs exert concentration-dependent biphasic effects on osteoblasts, promoting differentiation at low concentrations while inhibiting differentiation at high concentrations [15,32]. Against this background, therapeutic strategies aimed at restoring bone remodeling coupling have attracted increasing attention. Geranylgeraniol (GGOH) can bypass FDPS inhibition induced by BPs and directly provide GGPP to restore small GTPase prenylation, thereby re-establishing osteoclast and osteoblast function, promoting healing of jaw osteonecrosis, and reducing the risk of BRONJ recurrence [33,34,35]. Feldman et al. further demonstrated that incorporation of GGOH into bone cement particles restored osteoclast function and promoted wound healing in an MRONJ animal model, highlighting the therapeutic potential of local GGOH delivery for MRONJ treatment [36]. The vitamin K_2_ analog menaquinone-4 (MK-4) exerts preventive and therapeutic effects by counteracting osteoblast apoptosis through inhibition of SIRT1-dependent pathways [37]. These strategies target BRONJ through two distinct approaches, namely restoration of small GTPase prenylation and antagonism of osteoblast apoptosis, highlighting the translational potential of targeting bone remodeling coupling.

In summary, the effects of BPs on osteoclasts can be described as a “triple hit”: loss of the actin ring and sealing zone, mitochondrial dysfunction, and impairment of GTPase-mediated signaling and survival pathways. Together, these alterations result in osteoclast apoptosis and suppression of bone remodeling. However, as a central hub linking cholesterol biosynthesis and protein prenylation, inhibition of the mevalonate pathway generates metabolic alterations, including IPP accumulation and FPP/GGPP depletion, that transcend the boundary between skeletal and immune cells. The former acts as a phosphoantigen capable of activating γδ T cells, whereas the latter impairs signal transduction in multiple immune cell populations. The following sections will systematically discuss how these metabolic disturbances are amplified through distinct immune cell subsets and ultimately culminate in a local inflammatory storm within the jawbone microenvironment.

## 3. Systemic Remodeling of the Immune Cell Network by BPs and Its Synergistic Effects in BRONJ Pathogenesis

### 3.1. Roles of γδ T-Cell Exhaustion and Treg/Th17 Imbalance in BRONJ

γδ T cells, regulatory T cells (Tregs), and T helper 17 (Th17) cells all belong to the T-cell lineage but undergo distinct functional remodeling during BRONJ pathogenesis. γδ T cells exhibit a dynamic transition characterized by “short-term activation and long-term exhaustion”, whereas suppression of Tregs accompanied by expansion of Th17 cells results in a shift in the Treg/Th17 balance. Although these cell populations act through different mechanisms, they synergistically contribute to the collapse of osteoimmune homeostasis through independent effector pathways. The following sections systematically discuss the pathological roles of these three T-cell subsets in BRONJ from two perspectives: the skeletal regulatory functions and functional evolution of γδ T cells, and the imbalance between Treg and Th17 cells together with its osteoimmune consequences. Based on these mechanisms, T-cell-targeted therapeutic strategies are subsequently summarized.

The regulation of bone metabolism by γδ T cells depends on their functional status and involves both osteogenic and osteoclastic processes. In terms of osteogenesis, activated γδ T cells secrete matrix metalloproteinase-3 (MMP3), which cleaves membrane-bound semaphorin 4D (mSema4D) into its soluble form (sSema4D). Soluble Sema4D subsequently binds to Plexin-B1/2 receptors on osteoblasts and activates the mTOR pathway, thereby suppressing osteogenic differentiation. Ou L et al. and Liu L et al. reported that zoledronic acid (ZOL) treatment inhibited mSema4D expression and reduced osteogenic differentiation-associated mRNA expression as well as alkaline phosphatase (ALP) activity [38,39]. In contrast, Movila A et al. observed significantly elevated levels of both sSema4D and γδ T cells in MRONJ lesions and demonstrated that γδ T cells were capable of producing sSema4D in vitro [40].

With respect to osteoclast regulation, γδ T cells exert bidirectional effects depending on their activation status. On the one hand, activated γδ T cells suppress the differentiation of immature dendritic cells (iDCs) into osteoclasts through downregulation of receptor activator of nuclear factor-κB (RANK), c-Fos, and ATP6V0D2, while simultaneously inhibiting osteoclastogenesis through the secretion of IFN-γ. Zhu X et al. and Phalke SP et al. demonstrated that co-culture with activated γδ T cells reduced osteoclast numbers and increased IFN-γ levels [41,42]. Pappalardo A et al. further confirmed that this effect could be reversed by anti-IFN-γ antibodies [43]. On the other hand, unstimulated γδ T cells promote osteoclastogenesis through IL-6 secretion [41]. In addition, γδ T cells expanded by ZOL plus IL-2 can directly eliminate osteoclasts through CD16-mediated antibody-dependent cellular cytotoxicity (ADCC), representing an additional cytokine-independent mechanism [44].

The abundance and function of γδ T cells are subject to time-dependent biphasic regulation by BPs. Mechanistically, inhibition of the mevalonate pathway by BPs leads to IPP accumulation, which acts as a phosphoantigen recognized by the TCR and stimulates γδ T-cell expansion [45], thereby explaining the initial increase in cell numbers. However, prolonged exposure results in impaired proliferative capacity. Wang H et al. reported that γδ T-cell expansion decreased by more than 50% after 8 h of ZOL treatment, accompanied by marked IPP accumulation [46], suggesting that excessive IPP accumulation may impair cellular expansion. Neutrophils also participate in this exhaustion process. Inoue Y et al. demonstrated that neutrophils suppress γδ T-cell expansion through the release of hydrogen peroxide, serine proteases, and arginase I [47]. Clinical evidence further indicates that circulating γδ T-cell numbers are significantly reduced in long-term BP users [48], whereas short-term ZOL administration upregulates the exhaustion marker TIM3 [49], suggesting progressive evolution toward terminal γδ T-cell exhaustion.

Parallel to the dynamic changes observed in γδ T cells, BPs also profoundly influence the differentiation fate of CD4^+^ T cells. Naïve CD4^+^ T cells differentiate into functionally opposing Treg and Th17 subsets under distinct signaling conditions, and BPs disrupt this balance. The differentiation process is governed by both transcriptional and metabolic mechanisms. Th17 differentiation depends on the BATF/IRF4-STAT3-RORγt axis induced by TGF-β and IL-6 [50,51,52]. In contrast, Treg differentiation is driven by TGF-β-mediated induction of Foxp3 through the Smad2/3-Smad4 pathway [53,54], while RORγt and Foxp3 mutually antagonize each other [55]. From a metabolic perspective, Th17 cells primarily rely on glycolysis and fatty acid synthesis through the PI3K/Akt/mTORC1 pathway, whereas Tregs depend on fatty acid oxidation and oxidative phosphorylation [56,57]. Interference with the mevalonate pathway by BPs may preferentially impair Treg differentiation through metabolic disruption, thereby providing a molecular explanation for the Treg/Th17 imbalance.

With regard to bone regulatory functions, Th17 and Treg cells play fundamentally opposing roles in bone metabolism. Th17 cells are key drivers of inflammatory bone resorption. On the one hand, they directly express RANKL, which acts on osteoclast precursors to promote osteoclastogenesis. On the other hand, they secrete IL-17, which induces RANKL expression in synovial fibroblasts and osteoblasts, thereby amplifying the signaling network that promotes osteoclast formation [58,59]. More directly, Kikuta J et al. revealed through intravital multiphoton microscopy that Th17 cells can induce mature osteoclasts to transition from a motile non-resorptive state to a stationary bone-resorptive state through direct cell-to-cell contact [60], indicating that the pro-resorptive effects of Th17 cells extend beyond the RANKL pathway. In contrast, Treg cells exert bone-protective effects by suppressing osteoclast precursors through CTLA-4-mediated cell–cell interactions and by secreting anti-inflammatory cytokines such as TGF-β and IL-10 to inhibit osteoclast differentiation [61,62]. Furthermore, Baysal E et al. demonstrated that Treg cells can promote osteogenic differentiation of bone marrow mesenchymal stem cells through paracrine mechanisms [63].

The systemic disruption of this balance by BPs has been demonstrated in multiple studies. Kikuiri T et al. reported that mice treated with ZOL and dexamethasone (Dex) exhibited significantly increased Th17 cell populations, significantly reduced Treg cell populations, a markedly decreased Treg/Th17 ratio, and a substantially higher incidence of BRONJ [64]. Similarly, Li Y et al. found that zoledronic acid treatment significantly reduced CD4^+^CD25^+^ T cells and Foxp3 mRNA expression while increasing IL-17 mRNA expression in the peripheral blood of minipigs [65]. Collectively, these findings indicate that BPs disrupt the Treg/Th17 balance by suppressing Treg differentiation while promoting Th17 differentiation, thereby shifting the bone microenvironment toward a pro-inflammatory and pro-osteoclastogenic state.

Based on the pathological mechanisms described above, therapeutic strategies targeting T-cell subsets have achieved encouraging progress in recent years. With regard to γδ T-cell-related targets, Ou L et al. demonstrated that recombinant human Sema4D promoted osteoblast differentiation and reduced necrotic bone formation [38]. Liu L et al. further showed that Sema4D supplementation restored osteoclast-specific gene expression, reduced osteoclast apoptosis, and promoted osteogenesis [39], suggesting that restoration of Sema4D signaling may reverse γδ T-cell-mediated inhibition of bone formation. In terms of restoring the Treg/Th17 balance, Lemos JVM et al. reported that digoxin, acting as a RORγt inhibitor, alleviated MRONJ by suppressing Th17 differentiation, reducing lesion radiolucency, and decreasing the secretion of inflammatory cytokines including IL-17, TNF-α, and IL-6 [66]. Kikuiri T et al. further demonstrated that systemic administration of mesenchymal stem cells (MSCs) inhibited Th17 differentiation while promoting Treg expansion, effectively restoring the Treg/Th17 balance and correcting Treg depletion [64]. Taken together, these strategies target distinct aspects of T-cell dysregulation through two complementary approaches, namely restoration of Sema4D signaling and reconstruction of the Treg/Th17 balance, providing potentially translatable directions for immunomodulatory intervention in BRONJ.

### 3.2. Central Role of BP-Induced Macrophage M1/M2 Polarization Imbalance in BRONJ Pathogenesis

Macrophages are highly plastic immune cells whose phenotypic polarization is precisely regulated by microenvironmental signals. Upon exposure to classical inflammatory activators, such as lipopolysaccharide (LPS) and interferon-γ (IFN-γ), macrophages undergo polarization toward the M1 phenotype. In contrast, exposure to alternative activators, including IL-4, IL-13, and IL-10, promotes polarization toward the M2 phenotype [67,68,69]. These two phenotypes exhibit distinct and mutually restrictive functions. M1 macrophages secrete large amounts of pro-inflammatory cytokines and chemokines, including TNF-α, TNF-β, and IL-1β, thereby mediating inflammatory responses and tissue damage. In contrast, M2 macrophages produce MCP4, MDC, TARC, IL-13, CCL17, and CCL18, contributing to anti-inflammatory responses and tissue repair [70,71]. Therefore, the balance between M1 and M2 macrophages determines whether the local microenvironment progresses toward “injurious inflammation” or “reparative healing”.

M1 and M2 macrophages exert opposing effects on bone metabolism, influencing skeletal homeostasis through promotion of bone resorption and inhibition of bone formation or, conversely, suppression of bone resorption and stimulation of bone formation. M1 macrophages promote osteoclast differentiation and inhibit osteoblast differentiation through multiple mechanisms. Studies by Liang B et al. and Jia Q et al. demonstrated that M1 macrophages enhance osteoclast activity and differentiation through the secretion of TNF-α and IL-1β, as well as through activation of the epidermal growth factor receptor (EGFR) signaling pathway. Moreover, M1 macrophages themselves can serve as osteoclast precursors [72,73]. However, this pro-osteoclastogenic effect is time-dependent. Wu CS et al. found that short-term inflammatory stimulation induced by M1 macrophages promoted osteoclast proliferation, whereas prolonged exposure reduced osteoclast viability to 71%, suggesting the existence of a self-limiting mechanism underlying the osteoclastogenic effects of M1 macrophages [74]. With regard to osteogenesis, Toledano R et al. reported that co-culture of osteoblasts with M1 macrophages resulted in decreased alkaline phosphatase (ALP) activity and reduced expression of osteogenic genes [75]. Zhu LF et al. and He Y et al. further elucidated the underlying mechanisms, demonstrating that M1 macrophages inhibit osteoblast differentiation through paracrine activation of the TLR4/AP1 pathway and secretion of CXCL9, respectively, accompanied by significant downregulation of osteogenesis-related genes and proteins [76,77].

In contrast, M2 macrophages broadly promote bone formation while suppressing bone resorption. On the one hand, M2 macrophages directly stimulate osteoblast differentiation through the secretion of growth-promoting factors, including BMP-2, TGF-β, IGF-1, and VEGF, while simultaneously inhibiting osteoclast differentiation through cytokines such as IL-4 [78]. On the other hand, their osteoprotective effects involve multiple molecular pathways. Wang Z et al. demonstrated that M2 macrophages promote bone regeneration through the expression of periostin [79]. Li Z et al. reported that M2 macrophage-derived exosomes promote osteogenic differentiation and suppress adipogenic differentiation of bone marrow mesenchymal stem cells (BMSCs), with increased miR-690, IRS-1, and TAZ expression implicating the miR-690/IRS-1/TAZ axis [80]. Furthermore, Guo ZY et al. revealed an additional mechanism by which M2-derived exosomes suppress osteoclast differentiation, namely through delivery of the deubiquitinase CYLD, which inhibits STAT3 signaling and thereby counteracts the osteoclastogenic effects of RANKL [81].

The effects of BPs on macrophages involve at least three levels: direct suppression of cellular survival and function, impairment of migratory capacity, and forced polarization toward the M1 phenotype. First, BPs directly inhibit macrophage differentiation, viability, and survival. Munoz MA et al. demonstrated that macrophages internalize BPs and undergo apoptosis as a consequence of mevalonate pathway inhibition [82]. Patntirapong S et al. further quantified this effect, showing that BP treatment reduced macrophage viability by 45% and decreased CD68 expression by 50% [83]. Hoefert S et al. confirmed that ZOL inhibits the differentiation of THP-1 monocytes into macrophages in a dose-dependent manner and reduces macrophage activity [84]. Second, BPs impair macrophage migration. Hoefert S et al. demonstrated that BPs suppress macrophage migration through inhibition of nuclear factor κB (NF-κB) expression and blockade of migration receptor activation [85]. More importantly, BPs actively drive macrophages toward the pro-inflammatory M1 phenotype. Kaneko J et al. found that ZOL treatment increased IL-1β mRNA expression and M1 marker expression while promoting the expression of the NLRP3 inflammasome and its downstream effectors [86]. Weber M et al. further demonstrated that BP treatment significantly increased the expression of the M1 markers CD86 and inducible nitric oxide synthase (iNOS) (6.66% vs. 3.47%), whereas expression of the M2 markers CD163 and CD68 was markedly reduced. Consequently, the M1/M2 ratio (iNOS/CD163) was significantly elevated (0.22 vs. 0.09) [87]. At the molecular level, Zhang Q et al. and Zhu W et al. revealed that BPs may promote M1 polarization through increased IL-17 secretion, which enhances M1 polarization via the STAT1 pathway while simultaneously suppressing M2 polarization through the STAT6 pathway. In addition, the TLR4/NF-κB signaling pathway contributes to M1 polarization, collectively amplifying the imbalance between M1 and M2 macrophages from both directions [88,89].

Given the central role of M1/M2 imbalance in BRONJ pathogenesis, restoration of macrophage polarization homeostasis has emerged as an important therapeutic strategy. Rosiglitazone, a potent peroxisome proliferator-activated receptor-γ (PPARγ) agonist, has shown therapeutic potential in BRONJ through restoration of the M1/M2 balance. Soundia A et al. reported that ZOL treatment significantly increased the number of iNOS-positive cells in mice, whereas rosiglitazone reversed this effect and improved both osteonecrosis and bone exposure [90]. In addition, O-GlcNAcylation, a nutrient-sensitive post-translational protein modification, has emerged as a promising approach for suppressing M1 polarization. Through multiple mechanisms, including inhibition of MyD88-TRAF3 interaction, prevention of HIF-1α degradation, and activation of AMPK leading to mTOR suppression, O-GlcNAcylation effectively restrains M1 polarization and provides a novel molecular target for BRONJ treatment [91,92].

### 3.3. Pathological Mechanisms of Early Activation and Long-Term Exhaustion of NK Cells in BRONJ

Natural killer (NK) cells can be classified into two subsets according to the expression of CD56 and CD16a: NK1 (CD16a^+^) and NK2 (CD56^+^). These cells eliminate target cells through the release of perforin and granzymes and the secretion of cytokines such as IFN-γ and TNF-α [93,94,95]. The regulation of NK cells by BPs exhibits a biphasic pattern characterized by early activation followed by long-term suppression. The activation mechanism involves BP-mediated inhibition of FDPS, resulting in GGPP depletion, activation of caspase-1, and cleavage of pro-IL-18 and pro-IL-1β into their mature forms, which subsequently activate NK cells and promote IFN-γ production [96]. However, this activation does not translate into a bone-protective effect. Tseng HC et al. found that osteoclasts treated with ZOL exhibited marked resistance to NK cell-mediated cytotoxicity, while simultaneously enhancing the secretion of IL-6, IFN-γ, IL-18, MIP-1α, and MIP-1β by approximately 2- to 6-fold [97]. These findings suggest that the cytotoxic function of NK cells is effectively bypassed, causing them to act instead as amplifiers of pro-inflammatory cytokine production. Prolonged exposure to BPs, in contrast, results in inhibitory effects on NK cells. Mueller SK et al. demonstrated that ZOL suppresses NK cell expansion and degranulation responses in a dose-dependent manner [98]. Ivanyushko T et al. further reported that the total number of NK cells was significantly reduced in patients with BRONJ, accompanied by an increase in the NK1 subset and a decrease in the NK2 subset [99]. Because NK1 cells primarily exert cytotoxic functions, their relative expansion may aggravate early tissue injury. In contrast, NK2 cells mainly perform immunoregulatory functions, and their reduction may impair local microenvironmental regulation. Together, these alterations drive the transition of NK cells from early activation to long-term exhaustion, shifting their role from immune surveillance toward maintenance of a pro-inflammatory state and thereby contributing to the initiation and persistence of BRONJ.

### 3.4. Roles of Neutrophil Dysfunction and Inflammatory Amplification in BRONJ

Neutrophils (PMNs) are among the first innate immune cells to arrive at sites of inflammation. They contribute to pathogen clearance and tissue repair through phagocytosis, degranulation, neutrophil extracellular trap (NET) formation, and the induction of monocyte recruitment [100,101,102]. The effects of BPs on neutrophils involve two parallel yet opposing pathways. First, inhibition of the mevalonate pathway impairs their fundamental defensive functions. Mechanistically, depletion of GGPP, caused by mevalonate pathway inhibition disrupts the prenylation of small GTPases, including Rac2, Rap1A, and Rho family members, which are essential regulators of neutrophil migration, cytoskeletal remodeling, phagocytosis, and nicotinamide adenine dinucleotide phosphate (NADPH) oxidase activation [20]. Impaired Rac2 and Rap1A prenylation compromises assembly and activation of the NADPH oxidase complex, thereby reducing reactive oxygen species (ROS) production and antimicrobial capacity [103]. In parallel, defective Rho-family signaling interferes with actin polarization and directional migration, ultimately weakening neutrophil recruitment to sites of oral injury [104]. Kuiper JW et al. demonstrated that ZOL dose-dependently suppresses fMLP-mediated chemotaxis and NADPH oxidase activity, while simultaneously increasing neutrophil mortality and attenuating the survival-promoting effects of granulocyte colony-stimulating factor (G-CSF) [105]. Second, BPs induce NET formation through activation of the ATP-P2X7-IL-1β axis, thereby converting neutrophils into amplifiers of inflammation. However, excessive or persistent NET formation may become detrimental by promoting BRONJ progression. NET-derived components, including extracellular DNA, histones, and neutrophil proteases, directly damage oral epithelial cells and vascular endothelial structures, thereby aggravating local tissue injury. Meanwhile, NETs activate macrophages and enhance the production of pro-inflammatory cytokines, creating a positive feedback loop that sustains neutrophil activation and inflammatory amplification. Furthermore, NETs facilitate microthrombus formation and compromise local perfusion, ultimately impairing vascular repair and mucosal healing [106,107]. Bando K et al. found that LPS enhanced the inflammatory effects of BPs, resulting in increased expression of the NET marker citrullinated histone H3 (CitH3) as well as elevated levels of IL-1β and IL-18 [108]. Together, these two pathways place neutrophils in a functional paradox characterized by impaired chemotactic and antimicrobial activity on the one hand and NET-mediated inflammatory amplification on the other. Chadwick JW et al. reported that following pamidronate (PA) administration, ROS production in blood and oral samples decreased by 45.5% and 41.4%, respectively, whereas neutrophil chemotaxis was reduced by 61.4% and 49.1%, respectively [109]. These findings suggest that modulation of excessive neutrophil activation may represent a potential therapeutic strategy for BRONJ.

### 3.5. Immunopathological Significance of Systemic Dendritic Cell Suppression in BRONJ

Dendritic cells (DCs) serve as a critical bridge between innate and adaptive immunity. They capture, process, and present antigens to T cells and activate naïve T cells through three essential signals: TCR-peptide antigen recognition, co-stimulatory molecules, and cytokines [110,111,112]. The inhibitory effects of BPs on DC function encompass multiple stages, including differentiation, maturation, phagocytosis, migration, and antigen presentation. Brufsky A et al. reported that ZOL downregulated the expression of genes associated with DC differentiation and function, including CD1a, CD11c, and CD83, while upregulating CD80 expression [113]. Bringmann A et al. further demonstrated that ZOL dose-dependently inhibited the expression of maturation markers on monocyte-derived dendritic cells (moDCs), including CD80, CD86, and CD40, as well as their chemotactic migratory capacity, IL-12 secretion, and ability to induce cytotoxic T lymphocytes (CTLs), thereby suppressing TLR4-mediated DC activation [114]. Elsayed R et al. subsequently found that ZOL treatment significantly reduced DC numbers, impaired the expression of MHC class II molecules and the co-stimulatory molecules CD86 and CD83, and inhibited migration toward CCL19, ultimately suppressing T-cell proliferation and activation [115]. In addition, BPs can induce the expression of IPP in DCs, thereby activating γδ T cells [113]. Taken together, the systemic suppression of DC function by BPs results in impaired initiation of adaptive immune responses and loss of local immune surveillance, creating an immunological “vacuum” that favors the development of BRONJ.

### 3.6. Immunosuppressive and Pro-Osteoclastogenic Effects of Myeloid-Derived Suppressor Cells in BRONJ

Myeloid-derived suppressor cells (MDSCs) are composed of immature myeloid cells (IMCs) and are characterized by potent immunosuppressive activity. MDSCs suppress T-cell and NK-cell functions in a non-major histocompatibility complex (MHC)-restricted manner, promote Treg expansion through both cell–cell contact and cytokine-mediated mechanisms, and induce M2 macrophage polarization via the secretion of IL-10 [116,117]. BP treatment enhances the recruitment and activity of MDSCs, driving their functional transition from “immune regulation” toward “pro-inflammatory bone destruction”. Zhou Z et al. demonstrated that MDSCs activate NF-κB signaling through upregulation of Neat1, which subsequently activates the NLRP3 inflammasome and promotes the secretion of IL-1β, IL-6, and TNF-α, thereby enhancing osteoclast formation [118]. Thiyagarajan R et al. further revealed that MDSCs promote osteoclastogenesis through the CD38 pathway; notably, the osteoclastogenic capacity of MDSCs was enhanced in aged mice and was accompanied by an eightfold increase in CD38 mRNA expression [119]. In addition, Okawa H and Kondo T et al. found that replacement of residual N-BPs with hydroxymethylene diphosphonate-deformable nanoscale vesicles (HMDP-DNV) increased the expression of anti-inflammatory signature genes in MDSCs [120]. Collectively, these findings indicate that MDSCs act as a pathological hub linking immunosuppression and bone resorption in BRONJ. On the one hand, MDSCs weaken local immune surveillance through suppression of T-cell and NK-cell activity and induction of Treg expansion and M2 macrophage polarization. On the other hand, they directly promote osteoclastogenesis through the Neat1/NF-κB/NLRP3 and CD38 pathways. These dual activities establish MDSCs as a critical mediator coordinating immunosuppression and osteoclastic bone destruction during BRONJ pathogenesis.

## 4. Cascade Amplification of Inflammatory Signaling Pathways Induced by Bisphosphonates

### 4.1. Pro-Inflammatory and Anti-Osteogenic Effects of the IL-36α Signaling Pathway in BRONJ

Interleukin-36α (IL-36α), a member of the IL-1 cytokine superfamily, plays an important role in regulating both innate and adaptive immunity. Upon binding to the IL-36 receptor (IL-36R), IL-36α recruits the IL-1 receptor accessory protein (IL-1RAcP) to form a heterotrimeric signaling complex, which subsequently activates the MyD88/IRAK/TRAF6/TAK1 signaling cascade and ultimately induces NF-κB and MAPK pathway activation, thereby regulating the expression of inflammatory mediators [121,122]. In BRONJ, the biological effects of IL-36α extend beyond classical inflammatory regulation and directly involve suppression of collagen synthesis. Kim S et al. demonstrated that the expression of IL-36 family members was significantly increased in a murine BRONJ model, whereas Col1a1 expression was markedly reduced. Mechanistically, IL-36α activates the Erk signaling pathway, thereby inhibiting TGF-β-induced nuclear translocation of the Smad complex and suppressing the expression of type I collagen (Col1α1) and α-smooth muscle actin (α-Sma) [123]. Therefore, IL-36α exerts a “dual-hit” effect in BRONJ: on the one hand, it drives inflammatory responses through the MyD88/NF-κB/MAPK signaling axis; on the other hand, it suppresses collagen synthesis through the Erk/Smad pathway. In this manner, IL-36α directly couples inflammatory activation with impaired tissue repair, thereby contributing to the initiation and progression of BRONJ.

### 4.2. Dual Pro-Inflammatory and Pro-Cell Death Effects of the TNF-α/TNFR1 Signaling Axis in BRONJ

Tumor necrosis factor-α (TNF-α) exists in two forms, membrane-bound TNF (tmTNF) and soluble TNF (solTNF). Among these, solTNF primarily binds to TNF receptor 1 (TNFR1), leading to the formation of Complex I and Complex II. Complex I promotes cell survival and the production of pro-inflammatory mediators through activation of the NF-κB and MAPK signaling pathways, whereas Complex II induces programmed cell death [124,125,126]. In BRONJ, the pathological significance of the TNF-α/TNFR1 signaling axis lies in its ability to directly couple local inflammation with osteocyte death. Aguirre JI et al. proposed that, in the presence of systemic risk factors, oral inflammatory signals, particularly the TNF-α/TNFR1 pathway, promote programmed necrosis and apoptosis of bone cells [127]. Morita M et al. demonstrated that alendronate increased TNF-α expression in osteoclast precursor cells and promoted their conversion into TNF-α-positive macrophages [128]. Soma T et al. further reported elevated TNF-α expression in ZOL-treated ONJ mice, whereas genetic deletion of TNF-α significantly suppressed ONJ progression [129]. Collectively, these findings suggest that BPs induce TNF-α expression and shift TNFR1 signaling from a survival- and inflammation-oriented state mediated by Complex I toward a death-oriented state mediated by Complex II. Consequently, the TNF-α/TNFR1 axis directly links the inflammatory microenvironment to programmed bone cell death, thereby promoting the initiation and progression of BRONJ.

**Figure 3:**
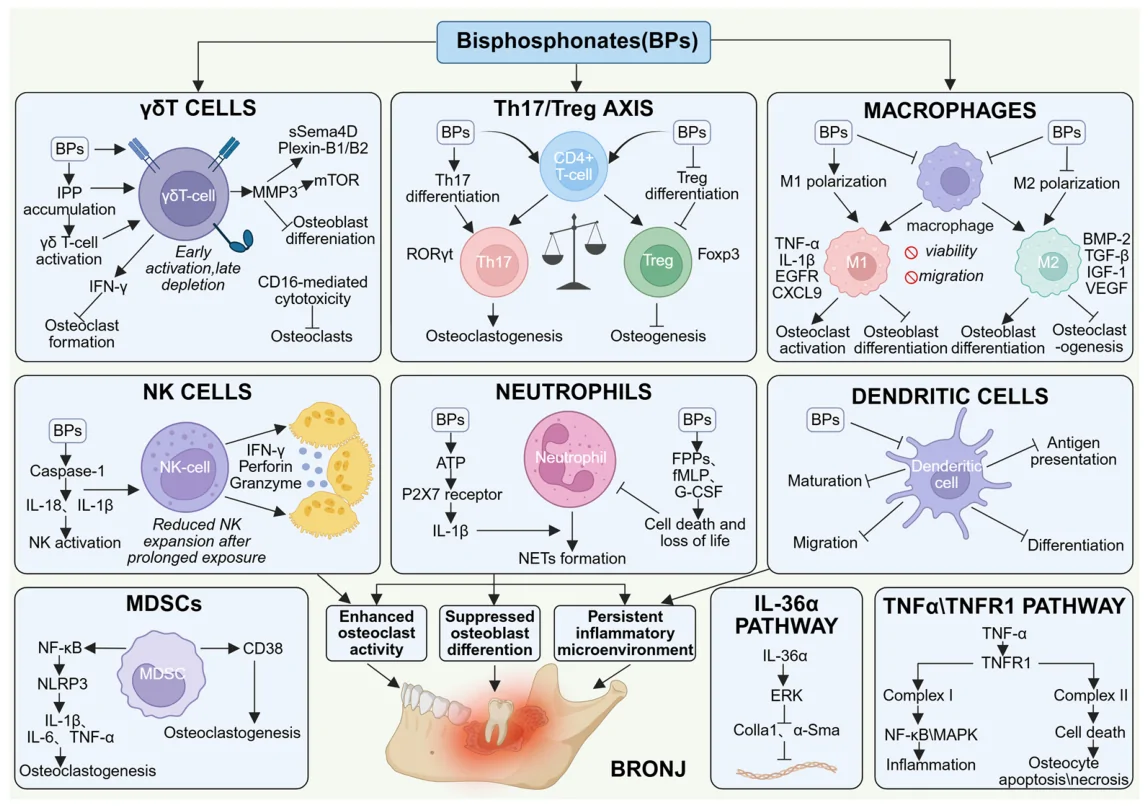
Detailed Immunological Cross-talk Map and Characterization of Multi-Cellular Dysregulation in the BRONJ Microenvironment. This schematic illustration summarizes the major immune-cell alterations and their interactions within the BRONJ microenvironment. Bisphosphonates (BPs) impair the function and abundance of γδ T cells, thereby compromising local immune surveillance, while suppressing regulatory T (Treg) cell expansion and disrupting immunoregulatory functions. In contrast, T helper 17 (Th17) cells undergo expansion and produce pro-inflammatory cytokines, including interleukin-17 (IL-17) and interleukin-22 (IL-22), which promote osteoclastogenesis and bone resorption. Myeloid-derived suppressor cells (MDSCs) contribute to immune suppression and modulate macrophage polarization, including promotion of M2-associated responses through IL-10. Meanwhile, the BRONJ microenvironment favors polarization toward the pro-inflammatory M1 macrophage phenotype, leading to increased production of inflammatory mediators such as tumor necrosis factor-α (TNF-α) and IL-1β and contributing to bone and soft-tissue damage. Created in BioRender. Pan, W. (2026) https://BioRender.com/1pyl2gb.

To comprehensively dissect the microenvironmental dynamics within the necrotic jaws, a multi-scale, detailed immunological cross-talk map is established to bridge macroscopic bone pathology with microscopic immunocellular dysregulation (Figure 3).

## 5. Synergistic Mechanisms of Angiogenesis Inhibition and Immune Microenvironment Remodeling in BRONJ

Inhibition of angiogenesis is one of the core mechanisms underlying BRONJ. However, its significance extends beyond the concept that ischemia and hypoxia simply lead to osteonecrosis. BP-induced vascular damage is closely coupled with immune microenvironment remodeling, and these two processes synergistically drive BRONJ progression through dysfunction of the endothelial–stromal–immune axis. BPs markedly inhibit angiogenesis both in vivo and in vitro, targeting multiple aspects of endothelial cell function [130]. Wood J et al. and Ziebart T et al. found that BPs dose-dependently inhibit the proliferation, migration, and adhesion of mature endothelial cells (HUVECs) and endothelial progenitor cells (EPCs), thereby reducing vascular sprouting in vitro [131,132]. Sharma D et al. found that BPs inhibit the differentiation of placental mesenchymal stem cells into the endothelial lineage and downregulate the expression of endothelial markers, including vWF and VEGF-R1/R2 [133].

The association between angiogenesis inhibition and the immune system is mainly mediated through three pathways. First, osteoclasts and osteoclast precursor cells participate in angiogenesis regulation. Under physiological conditions, osteoclasts stimulate angiogenesis through secretion of MMP-9, whereas osteoclast precursor cells promote angiogenesis through the SDF-1/CXCR4 pathway [134,135]. By inhibiting osteoclast differentiation, BPs simultaneously disrupt the pro-angiogenic signaling between osteoclast-lineage cells and endothelial cells. Ishtiaq S et al. found that BPs inhibit osteoblast secretion of VEGF (reduced by 34–39%) and ANG-1 [136]. Awad M et al. demonstrated that ZOL suppresses SDF-1 expression and reduces CXCR4 activity in osteoclast precursor cells, thereby inhibiting angiogenesis [134]. Second, angiogenesis is regulated through macrophage-derived extracellular vesicles. Shen X et al. found that macrophages treated with ZOL secrete extracellular vesicles carrying miR-149–5p, which inhibit endothelial cell proliferation, migration, and tube formation through the Rap1a/Rap1b/VEGFR2 pathway [137], directly linking alterations in macrophage immune status with vascular dysfunction. Third, BPs may impair the lymphatic system. Qin Z et al. found that ZOL induces endoplasmic reticulum stress and apoptosis in lymphatic endothelial cells (LECs) through the NAD^+^/SIRT6/XBP1s pathway, accompanied by a reduction in M1 macrophages within lymph nodes [138], suggesting that lymphatic injury may affect the regional trafficking of immune cells.

From a therapeutic perspective, platelet-derived growth factor-BB (PDGF-BB) promotes BRONJ healing by enhancing both angiogenesis and osteogenesis. Gao SY et al. demonstrated that treatment with a PDGF-BB-containing fibrin sealant resulted in near-complete restoration of both soft tissue wounds and bone tissue in a murine model [139]. Epidermal growth factor (EGF) has also been shown to partially reverse the inhibitory effects of ZOL on vascular endothelial cells through activation of the EGFR/Akt/PI3K signaling pathway [140]. These findings support the feasibility of therapeutic strategies based on the combination of pro-angiogenic stimulation and tissue repair promotion. However, whether such approaches can synergize with immune reconstitution remains to be elucidated.

## 6. Impairment Pathological Significance of Toxic Effects on Oral Soft Tissue Cells in BRONJ

The integrity of the oral mucosa depends on the proper function of normal human oral keratinocytes (NHOKs) and normal human oral fibroblasts (NHOFs). By inhibiting the mevalonate pathway, BPs induce senescence and apoptosis in both cell types. In addition, because the oral mucosa is in direct contact with the underlying bone surface and is simultaneously exposed to BPs released from both the circulation and bone tissue, this region is particularly susceptible to BP-induced toxicity [141]. Kim RH et al. demonstrated that BPs dose-dependently inhibit the proliferation of NHOKs and NHOFs and reduce their viability. Following treatment, the expression of phosphorylated P16 and P38 in NHOKs increased by 3.5-fold, accompanied by marked thinning of the epithelial layer [142]. Pabst AM et al. and Bullock G et al. further confirmed that N-BPs suppress the viability and migratory capacity of human oral keratinocytes (HOKs) and NHOFs, while reducing epithelial thickness in a dose-dependent manner [143,144]. Småland-Reksten A et al. reported that combined treatment with BPs and statins resulted in only 55% ± 9% wound closure in scratch assays, and that the secretion of IL-6, IL-8, G-CSF, MCP-1, and VEGF by NHOFs was significantly lower than that observed in control groups [145]. Taken together, these findings suggest a direct pathological link between soft tissue injury and osteonecrosis. BP-induced impairment of NHOKs and NHOFs, characterized by reduced proliferation, impaired migration, decreased metabolic activity, and diminished production of repair-associated factors, collectively contributes to delayed wound healing and failure of the oral mucosa to cover exposed bone surfaces. When combined with suppressed bone remodeling and impaired angiogenesis, these alterations create a triple pathological burden that ultimately drives the collapse of the local microenvironment in BRONJ.

## 7. Pathogenesis Clinical Evidence Revealing the Interaction Between the Immune Microenvironment and the Oral Microbiota in BRONJ

BP-induced immune impairment and reduced vascularization create favorable conditions for oral bacterial colonization. Oral microorganisms preferentially form biofilms on the surfaces of teeth and bone, thereby providing a persistent source of inflammatory stimulation [146,147]. In addition, certain saccharolytic bacterial species can establish hypoxic and acidic microenvironments that further exacerbate osteonecrosis following invasive dental procedures or odontogenic infections [148]. However, whether infection represents a cause or a consequence of BRONJ remains a matter of debate. Human microbiome studies have provided new perspectives on this issue and prompted a re-evaluation of the role of microbial communities in BRONJ pathogenesis.

Human studies have confirmed the enrichment of specific microbial taxa in BRONJ lesions. Yahara H et al. reported that the abundance of *Actinomyces* in the oral cavity of patients with BRONJ was 10.1% higher than that observed in healthy controls [149]. Choi NR et al. identified *Streptococcus* and *Tannerella forsythia* as dominant bacterial taxa in patients with MRONJ [150]. Li Q et al. further demonstrated that *Porphyromonas*, *Treponema*, and *Mogibacterium* enhance the pro-inflammatory effects of inflammatory mediators [151]. In addition, Badros AZ et al. found that patients with BRONJ exhibited reduced microbial diversity together with increased abundance of pathogenic *Streptococcus* species [152]. Animal studies have further validated the synergistic interaction between pathogenic microorganisms and BPs. Mawardi H et al. demonstrated that combined administration of PA and *Fusobacterium nucleatum* induced BRONJ-like lesions, whereas concomitant antibiotic treatment significantly inhibited disease development [153]. Wu S et al. reported that the combination of ZOL and *Porphyromonas gingivalis* suppressed bone regeneration and increased the expression of inflammatory genes [154]. Endo Y et al. further revealed that LPS enhances the soft tissue inflammatory effects of N-BPs, while inflammatory conditions promote the recruitment and accumulation of greater amounts of BPs within the jawbone [155].

Kalyan S et al. proposed the concept of “immune resilience”, suggesting that only a subset of individuals receiving N-BPs develop BRONJ because of interindividual differences in host immune reserve capacity. Transcriptomic analyses revealed significantly reduced expression of RANK, AHR, and FGF9 in patients with BRONJ, and these alterations may partially explain changes in microbial community composition [156]. In contrast to the enrichment of specific pathogens, commensal microbiota may play a protective role. Du W et al. found that depletion of commensal microbiota by antibiotics further aggravated osteonecrosis, and healing was worse in animals treated with both ZOL and antibiotics than in those treated with ZOL alone [157]. These findings suggest that the role of microorganisms in BRONJ is not simply that of infection, but rather the consequence of microbial dysbiosis in the context of an impaired immune microenvironment. They further indicate that indiscriminate antibiotic use may be counterproductive, whereas maintenance of microbial homeostasis may represent a new strategy for BRONJ management.

## 8. Role of Local Oral Factors in Triggering BRONJ

Tooth extraction is the most important risk factor for BRONJ. Schwech N et al. reported that the incidence of BRONJ following tooth extraction in patients with cancer receiving bisphosphonate therapy ranged from 11% to 50% [158]. Kizub DA et al. reported that tooth extraction accounted for 35.1% of BRONJ cases, followed by periodontal disease (24.6%) and denture-related trauma (10.5%) [159]. Dias MO et al. further confirmed that osteonecrosis of the jaw was the most common complication following tooth extraction in patients receiving bisphosphonate therapy [160]. Periodontitis exerts dual effects on BRONJ. Li CL et al. found that ZOL inhibited periodontitis-induced alveolar bone resorption but resulted in extensive areas of necrotic bone [161]. Aghaloo TL et al. demonstrated that sequestrum formation was observed only when ZOL treatment and periodontitis coexisted [162]. Williams DW et al. further revealed that periodontitis enhances RANKL signaling, thereby increasing the likelihood of necrotic bone exposure [163]. Apical periodontitis aggravates BRONJ through inflammatory pathways. Song M et al. found that pulp exposure promoted TNF-α expression and osteoclast differentiation, and tooth extraction following ZOL-induced periapical lesions significantly increased necrotic bone formation [164]. Kang B et al. further demonstrated that ZOL and apical periodontitis synergistically induced BRONJ, and that osteonecrosis occurred earlier than with bone exposure (84% vs. 33%) [165]. These findings indicate that local oral risk factors trigger BRONJ through two distinct mechanisms. Tooth extraction directly disrupts the bone-mucosal barrier, whereas periodontitis and apical periodontitis create a pro-inflammatory microenvironment through RANKL and TNF-α signaling, rendering BP-exposed bone tissue more susceptible to necrosis and exposure.

## 9. Cohort Studies Reveal That Systemic Diseases Increase BRONJ Risk Through Immune and Vascular Pathways

Epidemiological cohort studies have identified systemic diseases, including diabetes mellitus, rheumatoid arthritis (RA), and arterial hypertension, as independent risk factors for BRONJ [8,166]. Stelea CG et al. reported that the most common comorbidities were hypertension (38.9%), diabetes mellitus (22.2%), and previous chemotherapy (20.8%) [167]. Diabetes mellitus increases the risk of BRONJ through both immune and vascular pathways. Khamaisi M et al. found that the prevalence of diabetes was significantly higher in patients with BRONJ than in controls (58% vs. 12%), with the underlying mechanisms involving impaired neutrophil function, microvascular disease, and delayed wound healing [168]. Molcho S et al. further quantified that diabetes increased the risk of BRONJ by 2.78-fold, whereas microvascular complications, particularly diabetic nephropathy, increased the risk by 3.89-fold [169]. RA is itself characterized by chronic inflammation. Tenório JR et al. reported that RA increased the risk of BRONJ by 4.56- to 7.39-fold [170]. Taken together, systemic diseases synergize with BP-induced suppression of bone metabolism through two major pathways, namely immune dysfunction and microvascular impairment. These findings suggest that systemic comorbidities should be incorporated into BRONJ risk assessment.

## 10. Genetic Susceptibility to BRONJ Results from the Cumulative Effects of Variants in Multiple Pathways

Genetic susceptibility to BRONJ involves the cumulative effects of variants in multiple biological pathways, including immune function, bone metabolism, angiogenesis, and cellular homeostasis [171]. Candidate genes include *CYP2C8*, *SIRT1*, *ESR1*, *CYP19A1*, and *COL1A1* [172]. Furthermore, the *ATRAID* gene has been proposed as a potential susceptibility gene for MRONJ. Surface LE et al. identified *ATRAID* as an intracellular transporter involved in nitrogen-containing bisphosphonate uptake and FDPS-related signaling. Genetic variations in *ATRAID* may influence BP accumulation and the extent of mevalonate pathway inhibition, thereby contributing to interindividual differences in MRONJ susceptibility [173]. Kim S et al. identified *PDE4DIP*, *HLA-C*, and *HLA-B* as genes enriched with nonsynonymous variants through whole-exome sequencing [174]. Sarasquete ME et al. found that the *CYP2C8* rs1934951 homozygous genotype was present in 75% of ONJ cases, compared with 20% of controls [175]. Such E et al. reported a disease risk of 53% in homozygous carriers and 35% in heterozygous carriers [176]. Yang G et al. found that the A allele of *SIRT1* rs932658 reduced the risk of MRONJ by approximately 65% [177], and Bojtor B et al. likewise reported a significantly lower frequency of this variant in patients with MRONJ [178]. Choi SY et al. identified three SNPs within *ESR1* that were associated with an increased risk of MRONJ [179]. Nicoletti P et al. found that carriers of *RBMS3* rs17024608 had a 5.8-fold higher risk than non-carriers [180]. Kim JS et al. reported significant associations between MRONJ and *PPARGC1A* variants rs1151999 and rs294638 [181]. Yang G et al. further found that rs72817334 (OR = 2.27), rs11948737 (OR = 2.76), and rs2736308 (OR = 2.74) were associated with MRONJ risk and were involved in the regulation of immune- and inflammation-related genes, including *FDFT1*, *BLK*, and *CTSB* [182]. Arduino PG et al. identified an association between *VEGF* gene variants and MRONJ, suggesting a genetic contribution of the angiogenesis pathway [183].

Current evidence supports a polygenic susceptibility model of BRONJ involving not only immune, bone metabolic, and angiogenic pathways, but also genes regulating bisphosphonate transport and intracellular target engagement. The goals of genetic screening are to identify high-risk individuals for optimized BP treatment decisions, strengthen oral surveillance and preventive measures in patients carrying high-risk genotypes, and promote individualized and precision-based risk management of BRONJ in clinical practice. A consolidated overview mapping the pathological roles, associated genomic loci, and risk magnitudes of these diverse multi-pathway genetic variations in BRONJ is structured in Table 1.

## 11. Conclusions and Perspectives

Bisphosphonates contribute to the development of osteonecrosis of the jaw through multiple mechanisms, including osteoclast inhibition, impaired bone remodeling, angiogenesis suppression, soft tissue toxicity, and immune dysregulation. Among these mechanisms, aberrant immune regulation is increasingly recognized as a central driver of disease pathogenesis. Bisphosphonates suppress the proliferation and chemotactic activity of γδ T cells, Tregs, neutrophils, dendritic cells, and MDSCs, while simultaneously promoting Th17-cell expansion and macrophage polarization toward the M1 phenotype. Collectively, these alterations establish an immune microenvironment characterized by excessive inflammation and impaired tissue repair. More importantly, this immunopathological landscape extends beyond BRONJ. Recent studies have demonstrated that similar patterns of immune imbalance are widely present in osteoporosis, inflammatory arthritis, the bone tumor microenvironment, and metabolic bone diseases, suggesting that disruption of the osteoimmune axis may represent a common underlying mechanism of skeletal tissue injury. Therefore, using BRONJ as a representative model, this review seeks to establish a unified pathological framework for bone diseases centered on immune-inflammatory dysregulation and to provide new perspectives for precision immunotherapeutic strategies in skeletal disorders.

However, several limitations should be considered when interpreting the current mechanistic framework. First, the existing evidence is derived from heterogeneous experimental systems, including in vitro studies, animal models, observational cohorts, and a limited number of clinical investigations. Differences in experimental design, BP exposure conditions, disease models, and outcome evaluation may affect the consistency and translational relevance of individual findings. Therefore, further standardized experimental approaches and prospective clinical studies are required to validate these mechanisms. Moreover, although immune dysregulation, impaired angiogenesis, microbial imbalance, and soft tissue toxicity have been independently implicated in BRONJ pathogenesis, the relative contribution of each mechanism and their interactions remain largely unresolved. BRONJ is likely driven by a dynamic network involving osteoimmune regulation, vascular dysfunction, microbial alteration, and impaired tissue repair rather than a single dominant pathway. Future studies integrating multi-omics analyses, spatial transcriptomics, and longitudinal clinical data will be essential to identify key regulatory nodes. In addition, considerable clinical heterogeneity exists among patients receiving BPs, including variations in drug type, dosage, administration route, treatment duration, underlying diseases, dental procedures, comorbidities, and concomitant medications. These factors may influence individual susceptibility and restrict the generalizability of current conclusions. Future risk prediction models incorporating clinical characteristics, genetic background, immune status, and microbiome profiles may facilitate personalized prevention and management strategies.

## Figures and Tables

**Table 1:** Functional Impacts and Risk Magnitudes of Distinct Genetic Polymorphisms on BRONJ Susceptibility.

Gene	Impact	OR
*CYP2C8* rs1934951	promote	3.750
*SIRT1* rs932658	inhibit	0.351
*RBMS3* rs17024608	promote	5.800
*FDFT1* rs72817334	promote	2.270
*FDFT1* rs11948737	promote	2.760
*FDFT1* rs2736308	promote	2.740

## Data Availability

No new data were created or analyzed in this study. Data sharing is not applicable to this article.

## Abbreviations

The following abbreviations are used in this manuscript:

BPs	Bisphosphonates
BRONJ	Bisphosphonate-related osteonecrosis of the jaw
N-BPs	Nitrogen-containing bisphosphonates
non-N-BPs	Non-nitrogen-containing bisphosphonates
MRONJ	Medication-related osteonecrosis of the jaw
Treg	Regulatory T cell
Th17	T helper 17 cell
NK	Natural killer cell
MDSCs	Myeloid-derived suppressor cells
HAP	Hydroxyapatite
HMGCR	3-hydroxy-3-methylglutaryl-CoA reductase
IPP	Isopentenyl pyrophosphate
DMAPP	Dimethylallyl pyrophosphate
FDPS	Farnesyl diphosphate synthase
FPP	Farnesyl pyrophosphate
GGPP	Geranylgeranyl pyrophosphate
GGPPS	Geranylgeranyl pyrophosphate synthase
CoQ10	Coenzyme Q10
TCR	T-cell receptor
complex IV	Cytochrome c oxidase
RANKL	Receptor activator of nuclear factor κB ligand
TGF-β	Transforming growth factor-β
IGF-1	Insulin-like growth factor-1
GGOH	Geranylgeraniol
MK-4	Menaquinone-4
MMP3	Matrix metalloproteinase-3
mSema4D	Membrane-bound semaphorin 4D
sSema4D	Soluble semaphorin 4D
ZOL	Zoledronic acid
ALP	Alkaline phosphatase
iDCs	Immature dendritic cells
RANK	Receptor activator of nuclear factor-κB
ADCC	Antibody-dependent cellular cytotoxicity
Dex	Dexamethasone
MSCs	Mesenchymal stem cells
LPS	Lipopolysaccharide
IFN-γ	Interferon-γ
EGFR	Epidermal growth factor receptor
BMSCs	Bone marrow mesenchymal stem cells
NF-κB	Nuclear factor κB
iNOS	Inducible nitric oxide synthase
PPARγ	Potent peroxisome proliferator-activated receptor-γ
PMNs	Neutrophils
NET	Neutrophil extracellular trap
NADPH	Nicotinamide Adenine Dinucleotide Phosphate
G-CSF	Granulocyte colony-stimulating factor
CitH3	Citrullinated histone H3
PA	Pamidronate
ROS	Reactive oxygen species
DCs	Dendritic cells
moDCs	Monocyte-derived dendritic cells
CTLs	Cytotoxic T lymphocytes
IMCs	Immature myeloid cells
MHC	Major histocompatibility complex
HMDP-DNV	Hydroxymethylene diphosphonate-deformable nanoscale vesicles
IL-36α	Interleukin-36α
IL-36R	IL-36 receptor
IL-1RAcP	IL-1 receptor accessory protein
Col1α1	Type I collagen
α-Sma	α-smooth muscle actin
TNF-α	Tumor necrosis factor-α
tmTNF	Membrane-bound TNF
solTNF	Soluble TNF
TNFR1	TNF receptor 1
HUVECs	Mature endothelial cells
EPCs	Endothelial progenitor cells
LECs	Lymphatic endothelial cells
PDGF-BB	Platelet-derived growth factor-BB
EGF	Epidermal growth factor
NHOKs	Normal human oral keratinocytes
NHOFs	Normal human oral fibroblasts
HOKs	Human oral keratinocytes
RA	Rheumatoid arthritis
